# Biomarkers of Presbycusis and Tinnitus in a Portuguese Older Population

**DOI:** 10.3389/fnagi.2017.00346

**Published:** 2017-11-01

**Authors:** Haúla F. Haider, Marisa Flook, Mariana Aparicio, Diogo Ribeiro, Marilia Antunes, Agnieszka J. Szczepek, Derek J. Hoare, Graça Fialho, João C. Paço, Helena Caria

**Affiliations:** ^1^ENT Department, Hospital Cuf Infante Santo, NOVA Medical School, Lisbon, Portugal; ^2^Deafness Research Group, BTR Unit, BioISI, Faculty of Sciences, University of Lisbon (FCUL), Lisbon, Portugal; ^3^Faculty of Sciences, University of Lisbon, Lisbon, Portugal; ^4^Centro de Estatística e Aplicações, Faculty of Sciences, University of Lisbon, Lisbon, Portugal; ^5^Department of Otolaryngology, Charite University Hospital, Berlin, Germany; ^6^NIHR Nottingham Biomedical Research Centre, Division of Clinical Neuroscience, School of Medicine, University of Nottingham, Nottingham, United Kingdom; ^7^ESS/IPS- Biomedical Sciences Department, School of Health, Polytechnic Institute of Setubal, Setubal, Portugal

**Keywords:** presbycusis, *GRM7*, *NAT2*, tinnitus, markers, comorbidities

## Abstract

**Introduction:** Presbycusis or age-related hearing loss (ARHL) is a ubiquitous health problem. It is estimated that it will affect up to 1.5 billion people by 2025. In addition, tinnitus occurs in a large majority of cases with presbycusis. Glutamate metabotropic receptor 7 (*GRM7*) and *N*-acetyltransferase 2 (*NAT2*) are some of the genetic markers for presbycusis.

**Objectives:** To explore patterns of hearing loss and the role of *GRM7* and *NAT2* as possible markers of presbycusis and tinnitus in a Portuguese population sample.

**Materials and Methods:** Tonal and speech audiometry, tinnitus assessment, clinical interview, and DNA samples were obtained from patients aged from 55 to 75 with or without tinnitus. *GRM7* analysis was performed by qPCR. Genotyping of single nucleotide polymorphisms (SNPs) in *NAT2* was performed by PCR amplification followed by Sanger sequencing or by qPCR.

**Results:** We screened samples from 78 individuals (33 men and 45 women). T allele at *GRM7* gene was the most observed (60.3% T/T and 33.3% A/T). Individuals with a T/T genotype have a higher risk for ARHL and 33% lower risk for tinnitus, compared to individuals with A/A and A/T genotype, respectively. Being a slow acetylator (53%) was the most common *NAT2* phenotype, more common in men (55.8%). Intermediate acetylator was the second most common phenotype (35.9%) also more frequent in men (82.6%). Noise exposed individuals and individuals with ‘high frequency’ hearing loss seem to have a higher risk for tinnitus. Our data suggests that allele AT of *GRM7 c*an have a statistically significant influence toward the *severity* of tinnitus.

**Conclusion:** For each increasing year of age the chance of HL increases by 9%. The risk for ARHL was not significantly associated with *GRM7* neither *NAT2*. However, we cannot conclude from our data whether the presence of T allele at *GRM7* increases the odds for ARHL or whether the A allele has a protective effect. Genotype A/T at *GRM7* could potentially be considered a biomarker of tinnitus severity. This is the first study evaluating the effect of *GRM7* and *NAT2* gene in tinnitus.

## Introduction

Presbycusis [age-related hearing loss (ARHL)] is a universal feature of mammalian aging in which the auditory function is compromised, hearing thresholds increase, and frequency resolution gets poorer. As a result, in noisy environments speech-understanding deteriorates and temporal processing deficits in gap detection measures increase (Lee, 2013). In humans, this condition affects tens of millions of people world-wide (Yamasoba et al., 2013). Many people with hearing loss also experience tinnitus, which is the perception of a sound in one or both ears or in the head in the absence of an external sound source (Jastreboff and Hazell, 1993).

Presbycusis is complex in that it has repercussions at a physical, cognitive, emotional, and social level; quality of life can deteriorate, and for some people presbycusis could lead to depression, social isolation and lower self-esteem (Lee, 2013; Ciorba et al., 2015). Environmental factors such as diet, physical exercise, smoking, and intake of medications are some of the extrinsic factors predisposing to presbycusis. There are several auditory structures affected by presbycusis, such as hair cells, *stria vascularis*, afferent spiral ganglion neurons and the central auditory pathways (Fuentes-Santamaría et al., 2013). Based on results of audiometric tests and temporal bone pathology, Schuknecht and Gacek (1993) and later modified by Nelson and Hinojosa (2003), classified presbycusis as either sensory (downslope audiometry and cochlear degeneration), neural (downslope audiometry and very poor speech discrimination, spiral ganglion and nerve fibers degeneration), metabolic (audiometry in a platform and strial atrophy), cochlear conductive (downslope audiometry and thickening and stiffening of basilar membrane), mixed (mixture of the above), or undetermined (none of the above) types.

Depending on the type and severity of the hearing loss, several options are available to reduce the hearing difficulties and consequently improve quality of life. When patients are appropriately fitted and motivated, hearing aids and cochlear implants (CIs) are the most commonly used devices for treating mild-severe presbycusis. Electric-acoustic stimulation and active middle ear implants may also be suitable solutions for treating presbycusis (Sprinzl and Riechelmann, 2010).

Biological markers are widely used in oncology, hematology and in other medical disciplines to diagnose or to monitor various diseases. In otology, biological markers are not yet widely used, but once identified, they could provide a means of determining the time-course or most effective treatment for an individual with presbycusis or tinnitus. Potential biomarkers include mutations in mitochondrial DNA, chromosomal mutations, state of chronic inflammation, presence of certain diseases associated with earlier onset or progression of presbycusis (e.g., diabetes, hypertension) and metabolic diseases (Van Eyken et al., 2007; Verschuur et al., 2014). It was recently estimated that 35–55% of auditory aging could have a genetic background (Ruan et al., 2014). Of interest are genes coding for glutamate receptors as glutamate is the main excitatory neurotransmitter in the peripheral and central auditory pathways. It has been suggested that increased release of glutamate may be involved in the auditory aging and the generation and maintenance of tinnitus by causing “excitotoxicity.” There are many types of glutamate receptors, such as *N*-methyl-D-aspartate (NMDA) and alfa-amine propionic acid (AMPA), the latter being the most relevant receptor in physiological neurotransmission at auditory pathways. NMDA receptors are not essential for the auditory transmission, but they have been shown to be expressed in the cochlea after induction of tinnitus. Moreover, it has been demonstrated that the application of NMDA antagonists directly into the cochlear fluid can block salicylate-induced tinnitus in animals (Figueiredo et al., 2008).

*GRM7* encodes a metabotropic glutamate receptor subtype 7 (mGluR7), a G protein-coupled receptor regulating auditory nerve excitability. When bound by L-glutamate, mGluR7changes the configuration of adenylyl cyclase, which has implications in the metabolism of AMPc, control of cellular cycle, and normal functioning of central nervous pathways. mGluR7 plays a general role in glutamate synaptic transmission (Voytenko and Galazyuk, 2011). In the auditory periphery, mGluR7 is thought to mediate glutamate excitotoxicity (Pujol et al., 1993) and in the cochlea mGluR7 maintains the glutamate-dependent equilibrium between the inner hair cells and the spiral ganglion neurons (Newman et al., 2012). Its role in the higher auditory pathways remains unclear (Lu, 2014). Single nucleotide polymorphisms (SNPs) of *GRM7* have been demonstrated to be associated with auditory aging in European (Friedman et al., 2009) and American populations (Newman et al., 2012) but not of a Chinese population (Luo et al., 2013). Interestingly, Newman et al. (2012) have reported that certain SNP variants of *GRM7* associate with poorer speech recognition in the elderly. The importance of *GRM7* in the auditory system is supported by the detection of mGluR7 in the inner and outer hair cells and in the spiral ganglion nerve (Friedman et al., 2009).

Highly concentrated glutamate may affect membrane permeability in the hair cells, causing an increase in Cl^-^ influx, and consequently an osmotic imbalance and membrane disruption (Puel et al., 1998). In addition, glutamate excitotoxicity induces apoptotic cell death and inflammation (Sahley et al., 2013). This was demonstrated in an animal model to be directly responsible for the loss of inner hair cells in a time-, dose- and tonotopy-dependent manner (Hu et al., 2015). Interestingly, neonatal exposure to monosodium glutamate has been shown to induce neuronal atrophy and dysmorphia in the cochlear nucleus and in the superior olivary complex (Foran et al., 2017). The physiological effects of glutamate excitotoxicity therefore are concluded to include ARHL (Pujol et al., 1993) and tinnitus (Brozoski et al., 2012; Sahley et al., 2013; Yu et al., 2016).

Oxidative stress represents an imbalance between the production of reactive oxygen species (ROS) and their detoxification and has been postulated to play a major role in the overall aging process and to significantly contribute to the ARHL. Oxidative stress in the inner ear, secondary to impairments in defense mechanisms caused by certain polymorphisms related to a battery of antioxidant systems, could make individuals more susceptible to ARHL (Seidman et al., 2002; Fujimoto and Yamasoba, 2014).

In the adult inner ear, presence of several detoxification and antioxidant enzymes including catalase, superoxide dismutase, glutathione peroxidase, and glutathione *S*-transferases (GST) has been demonstrated.

One of the sources leading to accumulation of ROS are insufficiently acetylated drugs which accumulate and may be converted into reactive drug metabolites by oxidative enzymes. *N*-acetyltransferase (*NAT*) are enzymes responsible for the detoxification of exogenic substrates via *N*-acetylation or *O*-acetylation. In humans, the catalytic activity by *NAT* isoenzymes *NAT1* and *NAT2* may be regulate by these substrate concentration. Both isoenzymes are highly polymorphic and catalyze many aromatic amines and hydrazine substances important for the balance of the oxidative status. In addition, *NATs* are known to be involved in the detoxification of harmful xenobiotics (Vatsis and Weber, 1993; Hein, 2002; Ünal et al., 2005b).

Variation in *NAT2* alleles or haplotypes resulting from combination of SNPs is responsible for the *N*-acetylation polymorphism. Regarding the latter, rapid, intermediate, and slow acetylator phenotypes have been demonstrated. These phenotypes are associated with the rate of catalytic activity and accordingly predispose toward drug toxicity (Rajasekaran et al., 2011).

Because the individuals with the null genotype for *NAT2* may be more susceptible to effects of environmental toxins and oxidative free radical cellular damage, the presbycusis becomes an ideal model for evaluation of gene-environmental interaction (Ünal et al., 2005a,b). Although many individuals have been exposed to several environmental risk factors, the ARHL develops to a different degree in various age groups. This suggests genetic host factor(s) contributing to the degenerative mechanisms (Ünal et al., 2005b).

Previous studies demonstrated the association between the common human *NAT2* alleles and ARHL. Independent studies have showed a significant association between *NAT2* polymorphisms and presbycusis, namely *NAT2^∗^6A* in the Turkish population (Ünal et al., 2005b) and in the European population (Van Eyken et al., 2007) with Caucasian subjects carrying a *NAT^∗^6A* mutant allele having an increased risk to Presbycusis (Bared et al., 2010). Other studies considering different *NAT2* alleles reported negative associations with ARHL (Dawes et al., 2015) and with the shape of the audiograms (Angeli et al., 2012), when considering audiometric patterns of presbycusis in older individuals. However, most authors suggested that *NAT2* gene is a susceptibility factor for development of hearing impairment (Ünal et al., 2005b; Dawes et al., 2015).

Here we explore the relationships between presbycusis, tinnitus, co-morbidities, and the genotypes of *GRM7* and *NAT2*, in a sample of older Portuguese adults.

## Patients and Methods

### Subjects

Inclusion criteria was the presence of sensory presbycusis, with or without tinnitus, in adults of any gender, aged between 55 and 75 years, from the Portuguese population.

Our sample included 78 older individuals (*n* = 45 women, *n* = 33 men).

For the purposes of inclusion presbycusis was defined as bilateral sensorineural deafness in downslope audiometric pattern, above 1000 Hz with poor speech discrimination (discrimination threshold > 40 dB SPL and 100% discrimination to 60 dB or worse). Although all included participants have presbycusis we will consider a subgroup with normal hearing because the adopted classification uses conversational frequencies.

Exclusion criteria were considered: inability to understand and sign the informed consent due to a significant cognitive impairment, an uncompensated medical disorder that requires urgent evaluation or if the individual has a serious psychiatric disorder. Also individuals over 55 years who presented possible factors that may overlap the variables under study were excluded [e.g., Ménière’s disease, chronic otitis media, otosclerosis, tinnitus from disease of the outer ear (occlusive exostosis, outer otitis)], history of ototoxic drugs use, massive noise exposure, a history of previous malignancy with chemotherapy, history of autoimmune disorders and neurodegenerative and demyelinating diseases.

This study had the approval of the Ethical Committees from Hospital Cuf Infante Santo (November 26th, 2014), Nova Medical School (n°65/2014/CEFCM) and the National Department of Personal Data Protection (authorization number:1637/2016).

Accordingly we obtained the Institutional Scientific Review Board approval of the process for taking informed consent and overall study design. The study was conducted in accordance with the Declaration of Helsinki.

### Clinical Evaluation

Written informed consent, clinical and familial history, audiological evaluation and a blood sample, using Whatman^®^ FTA^®^ card technology, was obtained from every subject.

A questionnaire concerning epidemiologic data (demographic, previous and present diseases, toxicological habits and noise exposure) was completed by the researcher through participant interview.

#### Audiological Assessment

Hearing thresholds were determined by pure tone audiometry (air and bone) according to ISO 8253 and 389. The exam was performed in a soundproof booth using an Interacoustics^®^, Assens, Denmark audiometer (Model: AC40, Serial No.: 98 019 046) and TDH39 headphones fitted with noise-excluding headset ME70 and bone conductor B-71. Audiometry was performed at frequencies from 0.25 to 16 kHz (standard tonal audiometry and extended high frequency). The category of Hearing Loss (HL) was defined according to the average threshold across 500, 1000, 2000, and 4000 Hz in the better ear as mild (21–40 dB), moderate (41–70 dB), severe (71–95 dB) or profound (>95 dB), from an average of thresholds at 500, 1000, 2000, and 4000 Hz in the better ear, according to the European Working Group Genetics of Hearing Impairment (After Liu and Xu, 1994; Parving and Newton, 1995).

Speech audiometry evaluation was obtained with headphones (using mp3 player), or in open field, where the evaluator was hiding his lips to prevent lip-reading. The number of disyllables that patient repeats correctly was recorded. This intelligibility threshold for two-syllable words intends to measure hearing sensitivity threshold through the intensity level identification in which the patient can correctly identify 50% or more of a disyllables list. On the other hand, the speech discrimination evaluates the lowest intensity level at which a listener can understand speech.

#### Tinnitus Assessment

Psychoacoustic assessment consisted of loudness match, pitch match, minimum masking level (MML) or Feldmann masking curves, residual inhibition, and loudness discomfort levels (LDL). The severity of tinnitus was evaluated using the Tinnitus Handicap Inventory (THI; Newman et al., 1996). THI comprises 25 questions concerning tinnitus, and the response options are “Yes,” “Sometimes,” and “No,” respectively, corresponds to 4, 2, and 0, accounting for a total score that may vary between 0 and 100. The questionnaire comprehends three sub-scales or dimensions: Functional (11 items – contributing 0–44 for the final score), Emotional (9 items – contributing 0–36 for the final score) and Catastrophic (5 items – contributing 0–20 for the final score). This allow to verify which are the most affected aspects and accordingly choose the therapeutic interventions. The total score of the responses allows tinnitus classification according to its severity or impact in daily life – 0–16: Slight or no handicap (Grade 1), 18–36: Mild handicap (Grade 2), 38–56: Moderate handicap (Grade 3), 58–76: Severe handicap (Grade 4), 78–100: Catastrophic handicap (Grade 5).

Additionally THI is a self-administered instrument, easy to quote, to interpret and has good psychometrics properties (McCombe et al., 2001).

### Genetic Analysis

Total genomic DNA was extracted from a blood sample on FTA cards using a commercial NZY Tissue gDNA Isolation Kit (NZYTech, Lisbon, Portugal), strictly according to the manufacturer’s instructions. Molecular analysis of *GRM7* gene was assessed by qPCR for A/A, A/T, and T/T genotypes, at rs11928865 SNP. Concerning *NAT2* gene, rs1041983, rs1801280, rs1799929, rs1799930, rs108 and s1799931 were assessed by qPCR or by bidirectional sequencing of the target region in order to identify all the SNPs.

### Statistical Analysis

We conducted a descriptive analysis for variables such as gender and age. The audiograms were analyzed considering the best ear (estimated based on the lowest average of frequencies of 0.5–4 kHz). We also evaluated the “high frequency” pure-tone average (PTA) at 2, 4, and 8 kHz (Newman et al., 2012). Chi-square Test or Fisher Exact Test for general association between two variables were used. Mann–Whitney or Kruskal–Wallis (for more than two groups) tests were employed to compare hearing thresholds. A Dunn’s test with a Bonferroni correction was applied for multiple pairwise comparisons. The level of significance considered was *p* = 0.05.

All the results were analyzed through logistic regression model, where age and gender where considered as control for all other variables.

## Results

Participants in our study were 78 older adults aged 64.6 ± 5.58 years old (range = 55–75 years old). Most participants were female (*n* = 45, 57.7%), presenting an average age of 64.1 ± 5.35 years old. For men (*n* = 33, 42.3%), the mean age was 65.3 ± 5.89 years old (**Table 1**).

**Table 1:** Distribution of the individuals by subgroups according to hearing loss, tinnitus presence (PTA = Pure Tone Average) and gender.

Subgroup	Audiological characteristic	Gender		*n*
		Male	Female	
1	PTA ≤ 20 without Tinnitus	5	13	18 (28%)
2	PTA ≤ 20 with Tinnitus	8	15	23 (29.5%)
3	PTA ≥ 20 without Tinnitus	6	4	10 (12.8%)
4	PTA ≥ 20 with Tinnitus	14	13	27 (34.6%)

**Total**		33	45	**78**

### Hearing Thresholds

**FIGURE 1:**
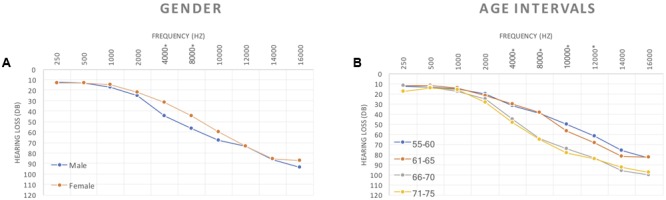
Tonal audiogram curves showing differences between: **(A)** gender and **(B)** age intervals, for all the frequencies used to estimate HL status.

The average hearing threshold values (by gender and age) are shown in **Figure 1**. There were significant differences between the gender groups regarding average and median values of hearing thresholds at the frequencies of 4 kHz (*p*-value = 0.007) and 8 kHz (*p* = 0.031) when comparing male and female (**Figure 1**). There were significant differences between age groups regarding the average and median values of hearing thresholds at frequencies of 4 kHz (*p* = 0.003), 8 kHz (*p* < 0.001), 10 kHz (*p* < 0.001) and 12 kHz (*p* < 0.001) when comparing the different age groups (**Figure 1**).

**FIGURE 2:**
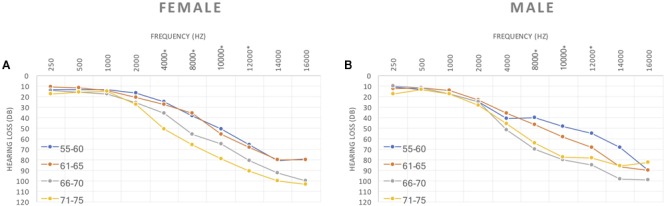
**(A)** Female and **(B)** Male hearing thresholds between the different aging groups.

When comparing hearing thresholds between the different age groups, we found significant differences in females at 4 kHz (*p* = 0.009), 8 kHz (*p* = 0.011), 10 kHz (*p* = 0.018) and 12 kHz (*p* = 0.002) (**Figure 2**). For males statistically significant differences were observed between age groups at 8 kHz (*p* = 0.009), 10 kHz (*p* = 0.003) and 12 kHz (*p* = 0.004) (**Figure 2**).

According to age and gender grouping and comparing males to females we found significant differences for hearing thresholds for the age group 55–60 years old for 1 kHz frequency (*p* = 0.022) and 4 kHz frequency (*p* = 0.028) (**Figure 2**).

**FIGURE 3:**
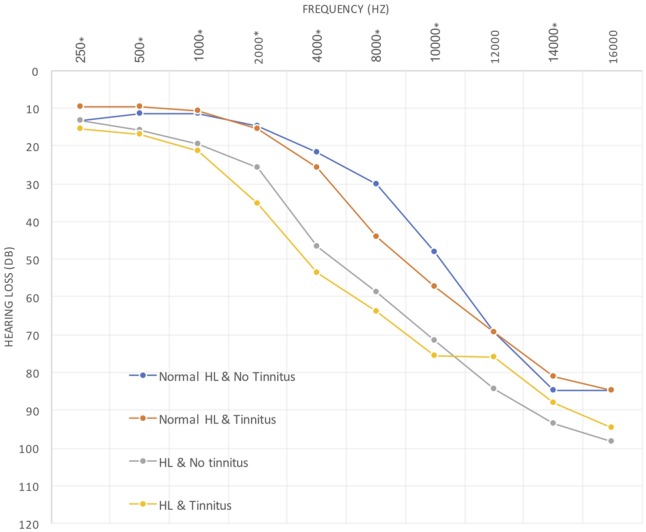
Hearing thresholds between subgroups.

Distribution of the individuals according to the hearing loss and tinnitus presence (**Table 1**) shows that in subgroup 1, 18 (23.1%) individuals who had normal hearing thresholds at speech frequencies (0.5–4 kHz) but not tinnitus; subgroup 2, 23 (29.5%) individuals who had normal hearing thresholds at speech frequencies and tinnitus; subgroup 3, 10 (12.8%) individuals who had hearing loss but not tinnitus; and subgroup 4, 27 (34.6%) individuals who had hearing loss and tinnitus (see also **Figure 3**). There are no statistical differences in age or gender between those four subgroups.

We found statistically relevant differences between the four described groups which corroborates the logical of having chosen this subdivision of our study population. (*p* < 0.001 at the majority of frequencies).

**FIGURE 4:**
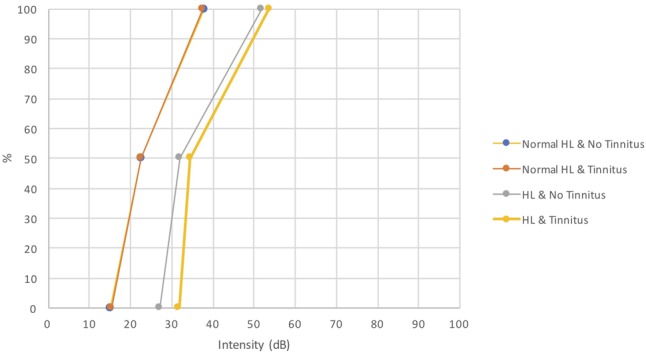
Speech audiograms between subgroups.

There were significant differences in speech audiograms (PTA, speech recognition threshold [SRT], 100%; *p*-value = <0.001; <0.001; <0.001, respectively) between subgroups, either for the right ear or for the left ear. The differences were found between subgroups 4 or 3 and the subgroups 1 and 2 for PTA (0%), SRT (50%) and (100%) (**Figure 4**).

Because our study population represents older adult individuals with sensory presbycusis we evaluated the “high frequency” pure-tone average (PTA) at 2, 4, and 8 kHz. We compared the groups of individuals with and without tinnitus and the four subgroups (**Table 1**). In respect to having or not tinnitus we found statistical differences between those groups (*p* = 0.003) (for more details see Appendix 1). We found statistically significant differences (*p* < 0.001) when comparing the four subgroups described in **Table 1** (for more details see Appendix 2).

Characterization of the considered comorbidities in our sample are presented in **Table 2**. Concerning hearing thresholds according to presence or not of the studied comorbidities, we found the following relevant significant differences: from 0.5 to 4 kHz for cholesterol; at 4 kHz for measles.

**Table 2:** Distribution of the most common comorbidities in the individual of the sample.

Comorbidities	*n*	
	Absent	Present
Cholesterol	29 (37.2%)	49 (62.8%)
Hypertension	43 (55.1%)	35 (44.9%)
Cardiovascular disease	73 (93.6%)	5 (6.4%)
Tinnitus	28 (35.8%)	50 (64.1%)
Diabetes	65 (83.3%)	13 (16.7%)
Thyroid problems	70 (89.7%)	8 (10.3%)
Smoking habits	44 (56.4%)	34 (43.6%)
Meningitis	77 (98.7%)	1 (1.3%)
Mumps	44 (56.4%)	34 (43.6%)
Measles	21 (26.9%)	57 (73.1%)
Tuberculosis	75 (96.2%)	3 (3.8%)
Ear diseases	62 (79.5%)	16 (20.5%)
Ear surgery	76 (97.4%)	2 (2.6%)
Noise exposure	51 (65.4%)	27 (34.6%)
Hormonal therapy	55 (70.5%)	23 (29.5%)
Ototoxic medication	58 (74.4%)	20 (25.6%)

When comparing the group of participants with and without tinnitus the most statistical relevant results were concerning ‘high frequency’ hearing loss and noise exposure. In our study population, 50 individuals (64.1%) had tinnitus.

**FIGURE 5:**
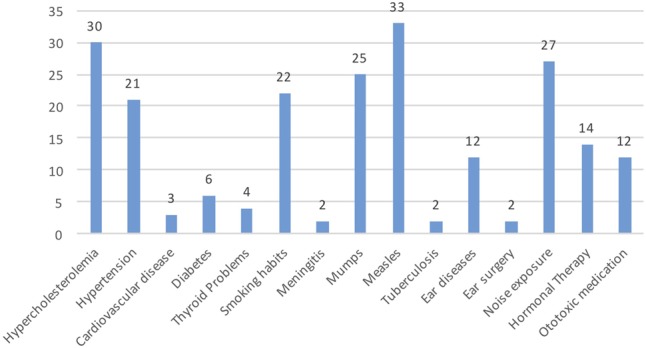
Distribution of the most common comorbidities in the individuals with tinnitus.

We have determined the distribution of studied comorbidities in our tinnitus population (**Figure 5**). The most prevalent were measles, hypercholesterolemia, noise exposure, mumps, smoking and hypertension, in a descendent order of frequency.

In our sample, 49 participants (62.8%) reported to have high blood values of cholesterol. Of those, 27 individuals (55.1%) were taking medication (statins) (Appendix 3). There was a significant association between tinnitus and statins intake in those individuals reporting hypercholesterolemia ($\hat{OR}$ = 0.28, p = 0.045, CI = 0.08 - 0.99) (**Table 3**). We found no relevant association between statins intake and hearing loss.

**Table 3:** Association between tinnitus and statins intake in individuals reporting hypercholesterolemia.

	Statins intake	Without Statins	$\hat{OR}$	*p*-value (fisher test)	95% IC
Tinnitus	13	17	0.28	*p* = 0.045	[0.08 – 0.99]
Without tinnitus	14	5			

*^∗^*p*-value < 0.05.*

#### Tinnitus Evaluation

**FIGURE 6:**
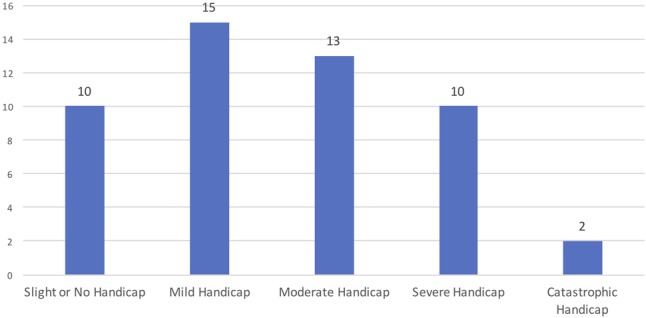
Distribution of the individuals according to THI.

Subgroups 2 and 4 included participants with tinnitus. Concerning tinnitus laterality, 33 of them reported to have a unilateral tinnitus (12 on the right ear and 21 on the left ear) and 17 participants have a bilateral tinnitus. According to THI score (**Figure 6**) for most participants tinnitus was bothersome, only 10 subjects had a slight handicap.

##### Modeling the data

All the results were analyzed through a logistic regression model age and gender were considered in all the models with the objective of controlling eventual confounding since these two factors are known to be related to hearing loss.

The regression logistic model was applied to HL considering female as reference (for more details see Appendix 4). The odds of developing presbycusis was significantly higher for males than for females ($\hat{OR}$ = 2.9,p = 0.032). When considering age as a covariate, the effect was slight but significant, being the odds of having hearing loss 9% higher for each increasing year ($\hat{OR}$ = 1.09,p = 0.03).

Using this statistical model for all the comorbidities considered and controlled for age the odds of having hearing loss was significantly lower for subjects with high cholesterol ($\hat{OR}$ = 0.33,p = 0.034).

We found no association between HL and high blood pressure or noise exposure.

In addition, using the regression logistic model for tinnitus considering men and the absence of tinnitus as reference, we found that noise exposure seems to influence the occurrence of tinnitus ($\hat{OR}$ = 3.65,p = 0.026, CI = 1.2 - 11.4), when considered isolated. This result is statistically very relevant.

There were no other statistically significant results concerning other comorbidities in this study (for more details see Appendix 5).

##### *GRM7* and *NAT2* genes

Results for *GRM7* gene at rs11928865 SNP refers to A or T alleles and contribute for three possible genotypes: A/A, A/T, or T/T. *GRM7* data are presented comparatively (**Table 4**) with data for Iberian Peninsula and Europe in order to compare our population with others.

**Table 4:** Comparative results for rs11928865 SNP on *GRM7* gene and comparison with other populations.

Genotypes	*N*	Frequency	Europe	United Kingdom	Iberian peninsula
A/A	5	0.064	0.087	0.055	0.065
A/T	26	0.333	0.382	0.473	0.393
T/T	47	0.603	0.531	0.473	0.542

**Total**	**78**	1	1	1	1

Some genetic specificity has been reported for different populations regarding deafness genes, and, interestingly, genotypes representativeness for the individuals of our sample were in accordance with values described in the European population as well as in the Iberian population.

Analyzing these results and considering the hearing thresholds, no significant differences were found in males or females when the three genotypes were compared, however, some differences in the pattern of the curves on the audiogram can be seen.

Considering the Tinnitus Handicap Inventory scores (please see **Figure 6**) the variable severe tinnitus (*n* = 12) joins the severe and catastrophic grades. We found relevant statistical association between the presence of *GRM7* and severe tinnitus (individuals having scored severe or catastrophic grade in THI). The results are present in the **Table 5**.

**Table 5:** Association between THI score (tinnitus severity) and *GRM7* gene.

*GRM7*	Severe tinnitus	*p*-value	Fisher test
A/A	1		
A/T	7	**0.0233^∗^**	**0.0175**
T/T	4		

*^∗^*p*-value < 0.05.*

We found no relevant statistical differences considering *GRM7* when comparing the four sub groups already described in **Table 1**, evidencing no relation with this SNP and the presence of presbycusis with or without tinnitus.

When considering genetic data, *GRM7* genotype was found not to be associated with the risk of developing presbycusis (*p* = 0.889). However, the odds of HL is higher in individuals presenting A/T (29%) or T/T (2%) genotype, than in A/A genotype. The same results were observed when controlling for age and gender, however, in this case the odds of HL in A/T genotype individuals was nearly 39% higher than for A/A genotype individuals. The odds of HL in T/T genotype was 15% higher than in A/A genotype.

The relation between tinnitus and GRM7 gene was evaluated considering two groups, one defined as “having an A allele” (AA + AT) other defined as “not having an A allele” (TT). Results were not significant ($\hat{OR}$ = 0.96) however, since the estimated ^ OR < 1, a decrease in the risk for tinnitus could be thought. The GRM7 genotype was not identified as a risk factor for tinnitus, neither when controlling for age ($\hat{OR}$ = 0.94) (OR = 0.94) for gender ($\hat{OR}$ = 0.93) or both simultaneously ($\hat{OR}$ = 0.93). Similar analysis was performed considering also two groups but defined as “having a T allele” (TT and AT genotypes). However, no significant association with tinnitus was found.

Genetic analysis of *NAT2* gene was performed in 65 individuals, 39 females (60%), and 26 males (40%). Rapid (R) phenotype was least common (12.3%, *n* = 8), followed by Intermediate (I) phenotype (35.4%, *n* = 23) and Slow (S) phenotype (52.3%, *n* = 34) (please see **Table 6**).

**Table 6:** Genotypes observed in the sample and their corresponding phenotypes.

Genotype	Phenotype
*NAT2^∗^4/NAT2^∗^5U; NAT2^∗^6A/NAT2^∗^6A; NAT2^∗^5B/NAT2^∗^5D; NAT2^∗^6J/NAT2^∗^13A; NAT2^∗^5A/NAT2^∗^5B; NAT2^∗^6N/NAT2^∗^6N; NAT2^∗^6A/NAT2^∗^6B; NAT2^∗^5D/NAT2^∗^5G; NAT2^∗^5B/NAT2^∗^5B; NAT2^∗^5R/NAT2^∗^12A; NAT2^∗^4/NAT2^∗^5J*	S
*NAT2^∗^4/NAT2^∗^4; NAT2^∗^12A/NAT2^∗^12C; NAT2^∗^4/NAT2^∗^12C*	R
*NAT2^∗^4/NAT2^∗^5B; NAT2^∗^4/NAT2^∗^6A; NAT2^∗^4/NAT2^∗^5B; NAT2^∗^4/NAT2^∗^5A; NAT2^∗^5B/NAT2^∗^12A; NAT2^∗^4/NAT2^∗^13A; NAT2^∗^4/NAT2^∗^5V; NAT2^∗^6A/NAT2^∗^13A; NAT2^∗^5B/NAT2^∗^13A*	I

The genotype 4/4 (considered as wild type) was observed in 9.1% (*n* = 6) of the individuals being the allele 4 present in 56.9% (*n* = 37) of the genotypes. The genotype 6A/6A previously associated with presbycusis was found in 6.2% (*n* = 4) of the individuals being the allele 6A present in 23.1% (*n* = 15) of the individuals. The most common genotype is 5B/5B accounting for 50% (*n* = 11) of all the homozygous genotypes (33.9%, *n* = 22) the sample (Kuznetsov et al., 2009).

We found no statistical differences in *NAT2* gene expression across our four subgroups described in **Table 1**, evidencing no relation with the presence of presbycusis with or without tinnitus. No significant association with ARHL was found, for the in the right and left ear or best or worst ear.

Considering the Tinnitus Handicap Inventory scores, we found significant association between severity of tinnitus (grades severe and catastrophic from THI) and the presence of *NAT2* gene (please see more details in the next sub-heading).

### Modeling the Data – *GRM7* and *NAT2*

All the results were analyzed through logistic regression model (**Tables 7, 8**) where age, gender and noise exposure were considered in the models with the purpose of controlling for confounding. The independent variable in the model was severe tinnitus (*n* = 12), (the sum of severe and catastrophic grades from THI) (**Figure 6**).

**Table 7:** Logistic regression model in the *GRM7* applied to severe tinnitus considering the genotype T/T as reference.

Variable^∗^	$\hat{OR}$	*p*-value (Wald test)	(95% IC)
*GRM7*			
A/A	2.9	0.443	(0.2, 42.2)
A/T	14.2	**0.009^∗∗^**	(2.0, 97.8)

*^∗∗^*p*-value < 0.05.*

**Table 8:** Logistic regression model in the *NAT2* applied to severe tinnitus considering intermediate acetylator as reference.

Variable^∗^	$\hat{OR}$	*p*-value (Wald test)	(90% IC)
*NAT2*			
Rapid acetylator	2.8	0.504	(0.4, 20.8)
Slow acetylator	5.7	0.095	(1.5, 21.9)

We have considered the genotype T/T because after crossing the *GRM7* gene with the tinnitus population, we found that the T/T genotype is more frequent and it is the most representative so it was chosen as the reference category.

The odds of developing severe tinnitus was significantly higher in the presence of genotype A/T when compared to genotype T/T ($\hat{OR}$ = 14.2, p = 0.009, CI = 2.0 -97.8). When considering the genotype A/A, no statistically significant difference was found ($\hat{OR}$ = 2.9, p = 0.443, CI = 0.2 - 42.2). The probability of severe tinnitus among individuals with genotype A/T is significantly higher when compared with individuals with the genotype T/T (for more details see Appendix 6).

When analyzing the presence of severe tinnitus through a logistic regression model considering *NAT2* as the independent variable and controlling for age, gender and noise exposure, the odds of developing severe tinnitus was significantly higher in the presence of slow acetylator phenotype when compared to intermediate acetylator ($\hat{OR}$ = 5.7, p = 0.095, CI = 1.5 - 21.9). No statistically significant difference was found with respect to rapid acetylator ($\hat{OR}$ = 2.8, p = 0.504, CI = 0.4 - 20.8) (for more details see Appendix 7).

## Discussion

In the present research, we conducted a case history questionnaire, hearing evaluation and gene screening analysis for *GRM7* and *NAT2* in a sample of patients aged between 55 and 75 years, in an attempt to find factors that might contribute to the diagnosis of presbycusis and tinnitus, which could be useful for diagnosis and future therapeutic interventions.

### Comorbidities Effect

Although in previous literature was described that individuals with thyroid problems present increased hearing thresholds, suggesting that thyroid hormones may act as regulators of the auditory system (Forrest et al., 1996) our results do not show any statistical relevance concerning this, one possible explanation is the sample size. Only 10% of our participants report thyroid problems which precludes statistical analysis.

Possibly for a similar reason our data doesn’t show that individuals with high blood pressure may be at greater risk of presbycusis than the normotensive. Hypertension has previously been associated with increasing of the hearing threshold (Agarwal et al., 2013, p. 614). Since both presbycusis and hypertension are common and widespread disorders, the fact that hypertension may influence presbycusis strongly suggests adding cardiologists to the multidisciplinary team of professionals screening for presbycusis and improving the quality of life of positively identified individuals (Agarwal et al., 2013).

Our results found that hypercholesterolemic individuals had a lower risk of HL, probably this is due to the fact that the majority of them (67%) were having medication (statins) to control cholesterol levels. These results are in accordance with previous publications (Gopinath et al., 2011). In individuals with hypercholesterolemia the chance of occurring tinnitus is 72% lower in those who have statins intake. According to our results It seems like the statins have a protector effect.

Noise exposure and “high frequency” hearing loss seems to influence the occurrence of tinnitus, those were two of the most statistical relevant findings in our study population, which is in accordance with previous literature (Hoffman and Reed, 2004).

### Gender and Age Effect

Significant differences on the HL degree were observed in different frequencies for different age groups (**Figure 2**). Our results show a significant age-dependent increase of hearing loss in about 13% for both genders, although the risk of developing presbycusis is about three times higher for men. This finding is consistent with a previous reports (Pearson et al., 1995) but contradicts another (Homans et al., 2016) where women were found to have more hearing loss.

According to our data, the risk of presbycusis increases 9% per year of life. Considering the increase in life expectancy of the population in industrialized countries, our result presents obvious consequences and must be considered for future clinical management guidelines.

In our sample tinnitus was present in 60.7% of the participants and men showed 53% more likelihood of developing tinnitus than women. This contradicts other results (Vielsmeier et al., 2012) who reported higher tinnitus prevalence in women but in a much younger population.

According to our data, and in agreement with previous literature (Hoffman and Reed, 2004; Shargorodsky et al., 2010) age is not associated with the risk of developing tinnitus.

### *GRM7* and *NAT2* Effect

We did not find a significant relationship between *GRM7* genotype and either presbycusis or tinnitus. Especially for men, some differences concerning the pattern in the audiogram curves were observed in relation to *GRM7* phenotypes. For both genders, the T allele in *GRM7* gene is the most common allele in our sample of older adults with presbycusis and tinnitus, where genotypes A/T and T/T present higher level of hearing loss compared to A/A genotype. Perhaps in a larger population it could be demonstrated that the allele A of *GRM7* plays a protective role in presbycusis.

Hence, according to our results, *GRM7* genotype does not seem to be predictive of presbycusis since the odds to have ARHL is not significant (*p* = 0.78). Corroborating our results, Luo et al. (2013) studying an all-male population found that the T-allele frequency was significantly different from the genotype A/A+A/T comparing ARHL patients and healthy controls and that the *GRM7* SNP A > T was significantly different between the two groups (Luo et al., 2013). On the other hand, our findings differ from Friedman et al. (2009) most likely due to sample size (Luo et al., 2013). Moreover, the impact of the other variables – environmental, lifestyle, noise exposure, cholesterol levels and stochastic element – perhaps has prevailed over the genetic factor, declining the importance between *GRM7* gene and ARHL. Certainly multicenter studies with higher sample sizes would overcome these aspects.

Concerning *NAT2* gene, Rapid (R) phenotype was the least common, followed by the Intermediate (I) and Slow (S) phenotypes.

We found relevant statistical association between the presence of the allele A/T of *GRM7* and severe tinnitus. The chance for having a severe grade of tinnitus (severe or catastrophic grades in THI) is 14,2 higher for those carrying the allele A/T compared to T/T. Probably in larger scale studies could be demonstrated the role of allele A/A that is the less frequent in our sample.

The odds of developing severe tinnitus was relatively higher in the presence of slow acetylator phenotype of *NAT2* when compared to intermediate acetylator.

Our data suggests that allele A/T of *GRM7* can have a statistically significant influence toward the *severity* of tinnitus. As well slow acetylator phenotype of *NAT2* seems to have a similar influence (not statistically relevant in our results). Nevertheless, those results should be interpreted with caution and future studies in larger scale are necessary to confirm this correlation.

However, present data shows that genotype A/T and T/T present, respectively, a 70 and 33.3% lower risk of developing tinnitus, when compared to A/A genotype. No other studies were found relating *GRM7, NAT2* and tinnitus.

## Conclusion

To the best of our knowledge, this is the first study on the association between *GRM7* and *NAT2* gene and the presbycusis and tinnitus in a population of Portuguese older adults.

Tinnitus was present in the majority of the presbycusis individuals.

Age and gender significantly influence the risk for presbycusis but not for tinnitus. Overall hearing thresholds rates increase exponentially with age (9% per year), and the increment rate and speed were gender-specific, but this increasing rate and velocity are different for women and men.

High blood pressure, thyroid diseases and hypercholesterolemia seem to have an effect on the hearing thresholds but no significant associations were found.

Our findings agree with previously observed correlations between tinnitus, noise exposure, and “high frequency” hearing loss.

No significant associations between presbycusis, tinnitus, and *GRM7* or *NAT2* were found in our sample. Our results precludes a definitive clarification about the role of *GRM7* as a possible genetic biomarkers for ARHL, although since the genotypes A/T and T/T have higher odds for HL than A/A genotypes, thus A allele could be pointed as protective biomarker for HL Nevertheless, the current state of knowledge regarding *GRM7* impact in presbycusis is insufficient to make conclusions, and so, further large-scale studies are necessary to clarify this relation.

Considering tinnitus severity (according to THI), our results bring-up very innovative conclusions.

Our data suggests the tracks that can lead to the pathway of a tinnitus severity biomarker. Potentially individuals carrying the allele A/T of *GRM7* and slow acetylator phenotype of *NAT2* (the later one with smaller statistic relevance) are prone to develop a more severe form of tinnitus, that requires specific therapeutic interventions and ideally personally tailored.

The occurrence of presbycusis is thought to be determined by genetic factors but can also be influenced by environmental or comorbidities effects, with a huge impact on quality of life and general health (Huang and Tang, 2010; Ciorba et al., 2015). However, there is still much research to explore and elucidate which risk factors contribute more to presbycusis and tinnitus, so this could help on therapeutic or preventive interventions (Huang and Tang, 2010).

Information on family history and clinical epidemiological data may help the design and development of future clinical management plans for an increasing presbycusis population.

## Author Contributions

HH conceived and designed this study and had contributions to all its stages. MAp and MA performed the statistical analysis. HH, MF, and HC contributed equally to all other stages of the manuscript development, drafted and revised the manuscript. DR worked with HH on interpretation of results and created appendices. DR created all audiometric figures. JP, MA, AS, DH, and GF provided consultative advice and revised the final manuscript.

## Conflict of Interest Statement

The authors declare that the research was conducted in the absence of any commercial or financial relationships that could be construed as a potential conflict of interest.
