# Supplementary material for: Biomarkers of Presbycusis and Tinnitus in a Portuguese Older Population
Source: Front Aging Neurosci. 2017 Nov 1;9:346. doi: 10.3389/fnagi.2017.00346 (PMC5672025; doi:10.3389/fnagi.2017.00346)
Supplement: Supplementary file 1 [file Data_Sheet_1.DOCX]

**Supplementary Material**

**Biomarkers of presbycusis and tinnitus in a Portuguese older population**

Haúla Haider*, Marisa Flook, Mariana Aparicio, Diogo Ribeiro, Marilia Antunes, Agnieszka J Szczepek, Derek J Hoare, Graça Fialho, João Paço e Helena Caria

*Correspondence: Corresponding Author: [hfhaider@gmail.com](mailto:hfhaider@gmail.com)

Appendix 1. High frequency (2, 4 e 8 Hz) in the hearing loss for tinnitus and no tinnitus population.

| *Variable* | Mean (SD) | T-test | p- value |
| --- | --- | --- | --- |
| *Better ear* |  |  |  |
| Tinnitus | 38.03 (16.20) | -3.0765 | 0.003 |
| No Tinnitus | 27.28 (13.71) |  |  |

* p-value<0.05

| *Variable* | Mean (SD) | T-test | p- value |
| --- | --- | --- | --- |
| *Left ear* |  |  |  |
| Tinnitus | 41.33 (16.14) | -3.117 | 0.0028 |
| No Tinnitus | 30.30 (14.03) |  |  |

* p-value<0.05

| *Variable* | Mean (SD) | T-test | p- value |
| --- | --- | --- | --- |
| *Right ear* |  |  |  |
| Tinnitus | 39.77 (17.28) | -3.1328 | 0.0026 |
| No Tinnitus | 28.70 (13.25) |  |  |

*p-value<0.05
