# Supplementary material for: Biomarkers of Presbycusis and Tinnitus in a Portuguese Older Population
Source: Front Aging Neurosci. 2017 Nov 1;9:346. doi: 10.3389/fnagi.2017.00346 (PMC5672025; doi:10.3389/fnagi.2017.00346)
Supplement: Supplementary file 2 [file Data_Sheet_2.DOCX]

**Supplementary Material**

**Biomarkers of presbycusis and tinnitus in a Portuguese older population**

Haúla Haider*, Marisa Flook, Mariana Aparicio, Diogo Ribeiro, Marilia Antunes, Agnieszka J Szczepek, Derek J Hoare, Graça Fialho, João Paço e Helena Caria

*Correspondence: Corresponding Author: [hfhaider@gmail.com](mailto:hfhaider@gmail.com)

Appendix 2. High frequency (2, 4 e 8 Hz) in the individuals by subgroups according to hearing loss and tinnitus presence.

| *Sub-groups* | PTA≤20 without Tinnitus | PTA≤20 with Tinnitus | PTA≥20 without Tinnitus | PTA≥20 with Tinnitus |
| --- | --- | --- | --- | --- |
| Better ear | 26.09  (8,86) | 26.09  (8.86) | 45.59  (12.56) | 48.21  (13.95) |
| Mean (SD) |  |  |  |  |
| Left ear | 22.69  (6.77) | 29.57  (10.57) | 45.56  (12.28) | 21.36  (13.03) |
| Mean (SD) |  |  |  |  |
| Right ear | 21.76  (6.4) | 27.39  (8.05) | 42.59  (12.56) | 50.31  (16.03) |
| Mean (SD) |  |  |  |  |

* p-value<0.05
