# Supplementary material for: Biomarkers of Presbycusis and Tinnitus in a Portuguese Older Population
Source: Front Aging Neurosci. 2017 Nov 1;9:346. doi: 10.3389/fnagi.2017.00346 (PMC5672025; doi:10.3389/fnagi.2017.00346)

**Supplementary Material**

**Biomarkers of presbycusis and tinnitus in a Portuguese older population**

Haúla Haider*, Marisa Flook, Mariana Aparicio, Diogo Ribeiro, Marilia Antunes, Agnieszka J Szczepek, Derek J Hoare, Graça Fialho, João Paço e Helena Caria

*Correspondence: Corresponding Author: [hfhaider@gmail.com](mailto:hfhaider@gmail.com)

Appendix 3. Description of individuals with hypercholesterolemia in our study


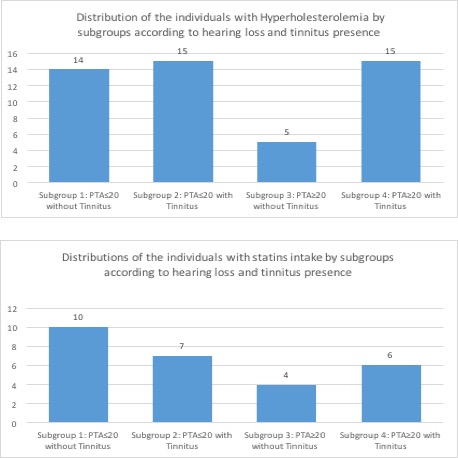

Supplement: Supplementary file 3 [file Data_Sheet_3.DOCX]
