# Supplementary material for: Biomarkers of Presbycusis and Tinnitus in a Portuguese Older Population
Source: Front Aging Neurosci. 2017 Nov 1;9:346. doi: 10.3389/fnagi.2017.00346 (PMC5672025; doi:10.3389/fnagi.2017.00346)
Supplement: Supplementary file 5 [file Data_Sheet_5.DOCX]

**Supplementary Material**

**Biomarkers of presbycusis and tinnitus in a Portuguese older population**

Haúla Haider*, Marisa Flook, Mariana Aparicio, Diogo Ribeiro, Marilia Antunes, Agnieszka J Szczepek, Derek J Hoare, Graça Fialho, João Paço e Helena Caria

*Correspondence: Corresponding Author: [hfhaider@gmail.com](mailto:hfhaider@gmail.com)

Appendix 5. Logistic model applied to Tinnitus considering men and the absence of tinnitus as reference

| Variable* | OR | p-value |
| --- | --- | --- |
| Sex |  |  |
| Male | 1.53 | 0.45 |
| Age | 1.01 | 0.84 |
