# Supplementary material for: Biomarkers of Presbycusis and Tinnitus in a Portuguese Older Population
Source: Front Aging Neurosci. 2017 Nov 1;9:346. doi: 10.3389/fnagi.2017.00346 (PMC5672025; doi:10.3389/fnagi.2017.00346)
Supplement: Supplementary file 6 [file Data_Sheet_6.DOCX]

**Supplementary Material**

**Biomarkers of presbycusis and tinnitus in a Portuguese older population**

Haúla Haider*, Marisa Flook, Mariana Aparicio, Diogo Ribeiro, Marilia Antunes, Agnieszka J Szczepek, Derek J Hoare, Graça Fialho, João Paço e Helena Caria

*Correspondence: Corresponding Author: [hfhaider@gmail.com](mailto:hfhaider@gmail.com)

Appendix 6. Logistic regression model in the GRM7 applied to severe tinnitus considering the genotype T/T as reference

|  | Estimate | Std. Error | z value | Pr(>\|z\|) |
| --- | --- | --- | --- | --- |
| (Intercept) | 2.19442 | 4.67151 | 0.47 | 0.63854 |
| GRM A/A | 1.06068 | 1.37056 | 0.774 | 0.43899 |
| GRM A/T | 2.65437 | 0.94913 | 2.797 | 0.00516 * |
| age | -0.05603 | 0.07318 | -0.766 | 0.44386 |
| gender F | -1.65257 | 0.89815 | -1.84 | 0.06577 |
| * p-value<0.05 | |  |  |  |
