# Supplementary material for: Biomarkers of Presbycusis and Tinnitus in a Portuguese Older Population
Source: Front Aging Neurosci. 2017 Nov 1;9:346. doi: 10.3389/fnagi.2017.00346 (PMC5672025; doi:10.3389/fnagi.2017.00346)
Supplement: Supplementary file 7 [file Data_Sheet_7.DOCX]

**Supplementary Material**

**Biomarkers of presbycusis and tinnitus in a Portuguese older population**

Haúla Haider*, Marisa Flook, Mariana Aparicio, Diogo Ribeiro, Marilia Antunes, Agnieszka J Szczepek, Derek J Hoare, Graça Fialho, João Paço e Helena Caria

*Correspondence: Corresponding Author: [hfhaider@gmail.com](mailto:hfhaider@gmail.com)

Appendix 7. Logistic regression model in the NAT2 applied to severe tinnitus considering Intermediate acetylator as reference

|  | Estimate | Std. Error | z value | Pr(>\|z\|) |
| --- | --- | --- | --- | --- |
| (Intercept) | -7.8823 | 5.16605 | -1.526 | 0.1271 |
| NAT2 S | 2.0182 | 1.00413 | 2.010 | 0.0444* |
| NAT2 R | 1.6963 | 1.50886 | 1.124 | 0.2609 |
| age | 0.0933 | 0.07558 | 1.235 | 0.2169 |
| gender F | -0.9486 | 0.84350 | -1.125 | 0.2608 |
| * p-value<0.05 | |  |  |  |
